# Isolation, characterization, and genome sequencing of a novel chitin deacetylase producing *Bacillus aryabhattai* TCI-16

**DOI:** 10.3389/fmicb.2022.999639

**Published:** 2022-09-12

**Authors:** Ying-yin Liang, Lu-qi Yan, Ming-hui Tan, Gang-hui Li, Jian-hao Fang, Jie-ying Peng, Kun-tai Li

**Affiliations:** ^1^Guangdong Provincial Key Laboratory of Aquatic Product Processing and Safety, Guangdong Provincial Engineering Technology Research Center of Seafood, Guangdong Province Engineering Laboratory for Marine Biological Products, College of Food Science and Technology, Key Laboratory of Advanced Processing of Aquatic Product of Guangdong Higher Education Institution, Guangdong Ocean University, Zhanjiang, China; ^2^Collaborative Innovation Center of Seafood Deep Processing, Dalian Polytechnic University, Dalian, China

**Keywords:** isolation, characterization, genome sequencing, *Bacillus aryabhattai* TCI-16, chitin deacetylase

## Abstract

Chitin deacetylase (CDA) is a chitin degradation enzyme that catalyzes the conversion of chitin to chitosan by the deacetylation of N-acetyl-D-glucosamine residues, playing an important role in the high-value utilization of waste chitin. The shells of shrimp and crab are rich in chitin, and mangroves are usually recognized as an active habitat to shrimp and crab. In the present study, a CDA-producing bacterium, strain TCI-16, was isolated and screened from the mangrove soil. Strain TCI-16 was identified and named as *Bacillus aryabhattai* TCI-16, and the maximum CDA activity in fermentation broth reached 120.35 ± 2.40 U/mL at 36 h of cultivation. Furthermore, the complete genome analysis of *B. aryabhattai* TCI-16 revealed the chitin-degrading enzyme system at genetic level, in which a total of 13 putative genes were associated with carbohydrate esterase 4 (CE4) family enzymes, including one gene coding CDA, seven genes encoding polysaccharide deacetylases, and five genes encoding peptidoglycan-N-acetyl glucosamine deacetylases. Amino acid sequence analysis showed that the predicted CDA of *B. aryabhattai* TCI-16 was composed of 236 amino acid residues with a molecular weight of 27.3 kDa, which possessed a conserved CDA active like the known CDAs. However, the CDA of *B. aryabhattai* TCI-16 showed low homology (approximately 30%) with other microbial CDAs, and its phylogenetic tree belonged to a separate clade in bacteria, suggesting a high probability in structural novelty. In conclusion, the present study indicated that the novel CDA produced by *B. aryabhattai* TCI-16 might be a promising option for bioconversion of chitin to the value-added chitosan.

## Introduction

Chitin, a linear polymer of N-acetyl-D-glucosamine, is widely distributed in the cuticle or exoskeleton of invertebrates, and the cell walls of algae and fungi (Das et al., 2016). It is considered that chitin is the second most abundant natural polysaccharide on earth, and has excellent potential for resource utilization. However, due to the highly extended crystalline structure, chitin is insoluble in water and organic solvents, leading to difficulties in its development and utilization (Kardas et al., 2012).

Chitosan, the critical N-deacetylated derivative of chitin, is water-soluble and possesses advantageous properties such as biocompatibility, biodegradability and non-toxicity (Tsigos et al., 2000; Kurita, 2006). To date, chitosan has been explored for considerable application in various fields, including recovery of impurities in water treatment, preparation of biomedical, pharmaceutical materials, and food additives (Fang et al., 2017; Yu et al., 2017; Xu et al., 2018; Zhang et al., 2018; Zhu et al., 2019). Currently, the production of chitosan from chitin is primarily obtained by means of thermochemical and physical processes (Ramos-Puebla et al., 2016). Nevertheless, these methods require a large amount of alkali and energy, which is costly and has the risk of environmental contamination. Moreover, physical or chemical means would cause random deacetylation of chitosan products, resulting in unstable product quality and low productivity (Chai et al., 2020). Therefore, it is desirable to replace the traditional physical or chemical means using more cost-effective, efficient and eco-friendly methods.

Chitin deacetylase (CDA, EC 3.5.1.41), a member of the carbohydrate esterase family 4 (CE4), can catalyze the conversion of chitin to chitosan by the deacetylation of N-acetyl-D-glucosamine residues (Lombard et al., 2014). Therefore, the CDA enzymatic deacetylation is regarded as a prospective tool for the production of chitosan, due to its many advantages such as mild reaction conditions, eco-friendliness, and uniform product deacetylation with few by-products (Subramanian et al., 2020; Ding et al., 2021). In past reports, a large number of fungi had been isolated and identified as the dominant CDA-producing microorganisms, such as *Mucor rouxii*, *Aspergillus nidulans*, *Absidia coerulea*, *Colletotrichum lindemuthianum*, *Saccharomyces cerevisiae*, *Scopulariopsis brevicaulis*, *Rhizopus nigricans*, and *Penicillium oxalicum* (Kafetzopoulos et al., 1993; Alfonso et al., 1995; Gao et al., 1995; Tsigos and Bouriotis, 1995; Martinou et al., 2002; Cai et al., 2006; Jeraj et al., 2006; Pareek et al., 2012).

The chitin-rich shells of crustacea such as crab and shrimp are one of the best available sources of chitin, but they are currently regarded as the waste materials of the food processing industries. Therefore, it is crucial to exploit efficient CDA specialized for the high-value utilization of chitin consisted in the shells of crab and shrimp. It is generally known that the coastal mangroves are the active habitat to shrimp and crab, and marine microorganisms have great possibility to produce unique and exceptional bioactive substances compared to terrestrial counterparts (Voser et al., 2022). In this study, a CDA-producing strain was screened from the mangrove mud and identified as *Bacillus aryabhattai* TCI-16, and its CDA-producing capability was subsequently investigated. Additionally, the whole genome of *B. aryabhattai* TCI-16 was sequenced and annotated to provide an insight into its chitin-degrading enzyme system responsible for chitin biodegradation.

## Materials and methods

### Media

Enrichment medium: chitin 10 g/L, (NH_4_)_2_SO_4_ 10 g/L, NaCl 0.5 g/L, K_2_HPO_4_ 0.7 g/L, KH_2_PO_4_ 0.3 g/L, MgSO_4_ 0.5 g/L, pH 7.0.

Selective medium: colloidal chitin 10 g/L, (NH_4_)_2_SO_4_ 10 g/L, NaCl 0.5 g/L, K_2_HPO_4_ 0.7 g/L, MgSO_4_ 0.5 g/L, agar 20 g/L, pH 7.20.

Differential medium: colloidal chitin 10 g/L, (NH_4_)_2_SO_4_ 10 g/L, NaCl 0.5 g/L, K_2_HPO_4_ 0.7 g/L, MgSO_4_ 0.5 g/L, p-Nitroacetanilide 0.2 g/L, agar 20 g/L, pH 7.20.

Luria Bertani (LB) medium: tryptone 10 g/L, yeast extract 5 g/L, NaCl 5 g/L, pH 7.50.

Fermentation medium: glucose 40 g/L, peptone 20 g/L, NaCl 0.5 g/L, K_2_HPO_4_ 0.7 g/L, KH_2_PO_4_ 0.3 g/L, CaCl_2_ 0.5 g/L, MgSO_4_ 0.5 g/L, pH 7.20.

All media were autoclaved at 121°C for 20 min before used. Chitin was purchased from Shanghai Yuanye Biotechnology Co., Ltd (Shanghai, China). Colloidal chitin was prepared as described previously (Pareek et al., 2013a). Briefly, 10 g chitin was completely dissolved in 200 mL HCl (32%, v/v) at room temperature. The solution was filtered with glass wool in pre-cooled distilled water until the dense white precipitate (colloidal chitin) was formed. After centrifuging at 10,000 rpm and 4°C for 10 min, the collected colloidal chitin was washed several times with distilled water until the pH reached neutrality, and then freeze dried.

### Isolation of chitin deacetylase-producing microorganisms

Soil samples were collected from the mangrove soil of Techeng Island in Zhanjiang, China. Each soil sample (5.0 g) was mixed in sterile water (50 mL) and incubated in enrichment medium at 170 rpm for 48 h at 30°C. Using the serial dilution and plating technique, an aliquot (0.1 mL) of the enriched culture was spread onto the colloidal chitin plate (selective medium) and cultured at 30°C for 48 h. Single colonies with transparent ring were picked out to spot inoculate on differential medium, with the uninoculated plate as control. After cultivation at 30°C for 48 h, the colony with the largest yellow circle was selected as a candidate CDA-producing strain.

### Identification of the chitin deacetylase-producing strain TCI-16

The isolated CDA-producing strain TCI-16 was identified based on its colonial morphology, Gram-staining capacity, cell morphology, and 16S rDNA gene sequence. Strain TCI-16 was spot inoculated on LB agar plates, and identified preliminarily by the colonial morphology, gram staining as well as scanning electron microscopy (SEM), in which SEM was performed as described previously (Ma et al., 2020). The DNA of strain TCI-16 was extracted using Sangon Bacterial Genomic DNA Kit (Shanghai, China) according to the manufacturer’s instructions. Then the 16S rDNA was amplified under the following PCR conditions: 95°C for 5 min; 30 cycles at 95°C for 1 min, 52°C for 30 s, 72°C for 1 min, and finally at 72°C for 10 min. The purified PCR products were sequenced and their sequencing data were subjected to phylogenetic analysis.

### Fermentation kinetics of *Bacillus aryabhattai* TCI-16 in shake flasks

*Bacillus aryabhattai* TCI-16 was grown on LB agar slant (15 × 150 mm) at 30°C for 48 h, and the fresh cell was washed with 10 mL sterilized water. The preculture was carried out in a 250 mL Erlenmeyer flask containing 50 mL LB medium inoculated with 1 mL suspended cell, and cultivated at 30°C on a rotary shaker at 170 rpm for 24 h. Then 2.5 mL preculture broth was inoculated into a 250 mL Erlenmeyer flask containing 50 mL fermentation medium, and the cultivation was performed at 30°C on a rotary shaker at 160 rpm for 72 h. The cultures were sampled every 6 h to measure the biomass, reserved sugar concentration (RSD) and CDA activity.

Cell biomass was measured by the dry cell weight (DCW), and the RSD was determined by the 3,5-dinitrosalicylic acid method (Miller, 1959). CDA activity was determined as described previously (Liu et al., 2016). Briefly, the fermentation broth was centrifuged at 8,000 rpm and 4°C for 20 min, and the collected bacterial cells were washed three times with phosphate-buffered saline (PBS, 10 mM, pH 7.0) and resuspended in the PBS solution. The cell suspensions were subsequently processed using ultrasonic on an ice bath and fragmented as follows: 200 W, on: 2 s, off: 3 s, 90 cycles. The cell-free extract obtained by centrifugation was used as a crude enzyme solution of CDA. For the enzymatic activity determination of CDA, 1 mL crude enzyme solution was mixed with 3 mL PBS (10 mM, pH 7.0) and 1 mL p-Nitroacetanilide (200 mg/L) in a 10 mL colorimetric tube, and then incubated at 55°C for 15 min. The enzymatic reaction was terminated by heating in a boiling water bath for 10 min, and the precipitate was removed by centrifuging at 5,000 rpm for 10 min. The supernatant was analyzed by measuring the absorbance at A400 in a UV-5800PC spectrophotometer. Heat-inactivated crude enzyme solution of CDA was used as negative control. One unit of CDA activity (U/mL) was defined as the amount of enzyme released from the reaction mixture of 1 μg of p-Nitroacetanilide per hour.

All tests were performed in triplicate, and the data were expressed as the mean ± standard deviation (SD). The significant differences between means were performed using ANOVA with Turkey’s test. Any difference was considered insignificant at *p* < 0.05. Origin Pro v8 was used to draw the figures.

### Whole genome sequence and functional annotation of *Bacillus aryabhattai* TCI-16

Whole-genome sequencing of *B. aryabhattai* TCI-16 was performed on the second-generation sequencing platform Illumina Hiseq × 10 platform using PE150 (pair-end) sequencing, in which the single-end sequencing read length was 150 bp. Each sample provided raw sequencing data with a coverage depth of not less than 100 × genomes, and finally assembled multiple genome scaffolds. Bioinformatics analysis was implemented using the data generated by the Illumina platform. All the data were uploaded on the Majorbio Cloud Platform (Ren et al., 2022)^1^ of Shanghai Meiji Biomedical Technology Co., Ltd.

Glimmer (Delcher et al., 2007) was used to predict the coding sequences of the bacterial genome. Protein-encoding genes were annotated with COG (Jensen et al., 2008) and GO databases, while the pathways were annotated using the KEGG databases (Kanehisa and Goto, 2000). Genome-wide rRNA and tRNA were predicted or scanned using Barrnap and tRNAs can-SE v2.0 (Chan and Lowe, 2019), respectively. Information about carbohydrate-active enzymes was obtained by the prediction software Diamond and annotated using the CAZy database (Lombard et al., 2014), based on which bioinformatics analysis of the genes coding chitin degrading enzymes were performed.

### Amino acid sequence analysis of chitin deacetylase in *Bacillus aryabhattai* TCI-16

The inserted sequence of CDA in the genome of *B. aryabhattai* TCI-16 was predicted and analyzed as follow: The physical and chemical properties of CDA were analyzed using the online software ProtParam^2^ (Gasteiger et al., 2005); Hydrophilicity of CDA was predicted using the tool of ProtScale (Kyte and Doolittle, 1982)^3^ (Gasteiger et al., 2005); Signal peptide of CDA was identified utilizing SignalP 4.1^4^ (Petersen et al., 2011); Transmembrane helix was predicted by TMHMM online service^5^ (Sonnhammer et al., 1998; Krogh et al., 2001); SOPMA software^6^ (Geourjon and Deleage, 1995) was used to elucidate the secondary structure of CDA; The structure prediction program SWISS-MODEL^7^ (Guex et al., 2009; Benkert et al., 2011; Mariani et al., 2013; Studer et al., 2014, 2020, 2021; Bertoni et al., 2017; Bienert et al., 2017; Waterhouse et al., 2018) was applied for 3D structure modeling; Blast search for the sequences of cDNA and protein of CDA gene was performed using BLAST software at the NCBI website^8^; Amino acid sequence alignments were carried out using ClustalW software (Combet et al., 2000), and phylogenetic tree was constructed using MEGA7 (Kumar et al., 2016) package based on the Neighbor-Joining method (Saitou and Nei, 1987).

## Results

### Isolation, characterization and identification of *Bacillus aryabhattai* TCI-16

**FIGURE 1:**
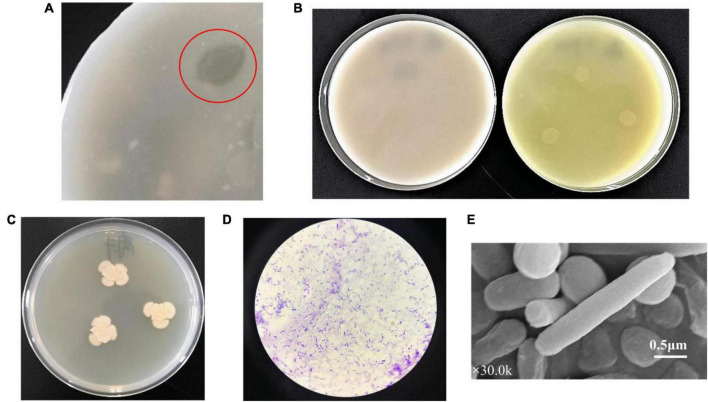
Morphological andfeatures of strain TCI-16. **(A)** After incubation at 30°C for 48 h, TCI-16 hydrolyzed colloidal chitin and formed transparent circles on the selective medium; **(B)** after incubation at 30°C for 48 h, CDA producing colonies reacted chromogenically and exhibited yellow circles in the differential medium (right), but the control was unchanged (left); **(C)** after incubation at 30°C for 48 h, colony morphology of the TCI-16 strain in LB medium; **(D)** gram staining; **(E)** SEM image (×30.0k).

After screening on the agar plates of selective medium, a strain TCI-16 with transparent circle was obtained from the mangrove soil (Figure 1A), suggesting that the strain had hydrolytic capacity for colloidal chitin. By further spot inoculation on differential medium, the strain TCI-16 presented an obvious color reaction in which the chromogenic p-Nitroacetanilide could be converted to the yellow p-Nitroaniline (Figure 1B), indicating that strain TCI-16 was a CDA-producing candidate. The colonies of strain TCI-16 on LB agar plates presented reddish yellow, rounded, translucent, and moist (Figure 1C). Cell morphology showed that strain TCI-16 was a rod-shaped and gram-positive bacterium (Figures 1D,E).

**FIGURE 2:**
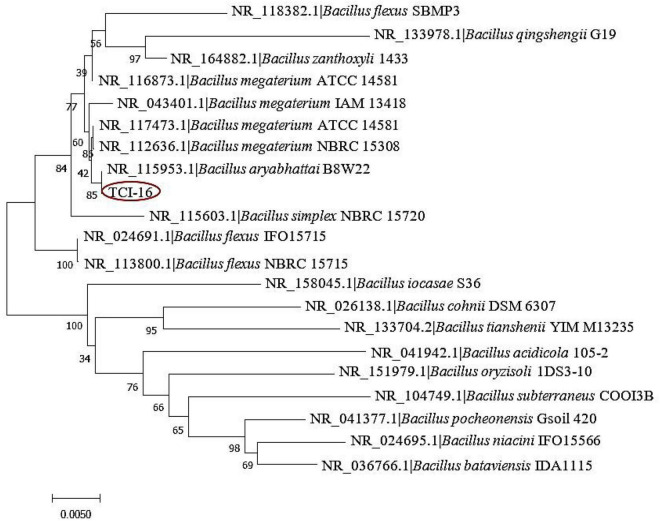
Phylogenetic tree of the strain TCI-16 based on 16S rDNA sequence.

Furthermore, the 16S rDNA gene sequence of strain TCI-16 was sequenced, and similarity analysis of sequence in Blast proved that the strain belonged to the genus *Bacillus*. The taxonomy of strain TCI-16 was confirmed by building a phylogenetic tree, which fell into one branch of *Bacillus aryabhattai* B8W22 (Shivaji et al., 2009) with 16S rDNA gene sequence similarity of 99.93% Figure 2. According to the morphological characteristics and 16S rDNA sequence analysis, the strain TCI-16 was identified and named as *Bacillus aryabhattai* TCI-16.

### Fermentation characteristics of *Bacillus aryabhattai* TCI-16

**FIGURE 3:**
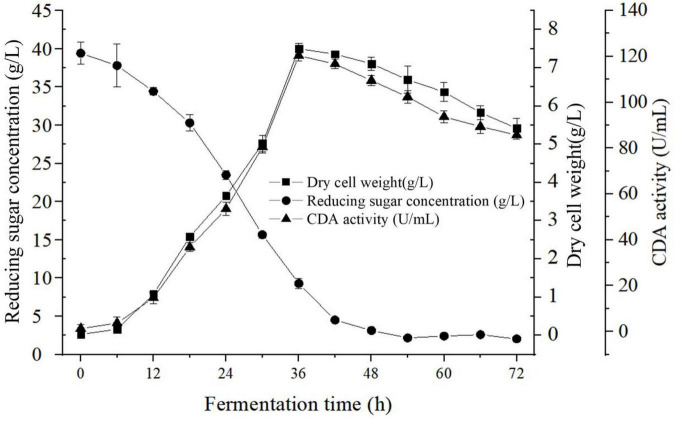
Fermentation characteristics curve of *Bacillus aryabhattai* TCI-16.

Figure 3 summarized the kinetics of cell growth, sugar consumption, and CDA activity during the fermentation processes of *B. aryabhattai* TCI-16. As shown in Figure 3, *B. aryabhattai* TCI-16 entered the logarithmic growth phase after 6 h, reaching a maximum DCW (7.50 ± 0.15 g/L) at 36 h. During the logarithmic growth period (6–36 h), the enzyme activity of the CDA gradually increased to the maximum value of 120.35 ± 2.40 U/mL at 36 h. Meanwhile, rapid sugar consumption was accompanied by the rapid cell growth and CDA production during the logarithmic growth period. Due to lack of carbon source for cell metabolism, *B. aryabhattai* TCI-16 fell into the stage of cell autolysis at 42 h, and the enzyme activity of CDA decreased accordingly. In conclusion, the kinetic curves of cell growth and CDA activity showed a good linear behavior, indicating that the produced CDA belonged to the typical growth-coupled product.

### Whole genome sequencing of *Bacillus aryabhattai* TCI-16

**FIGURE 4:**
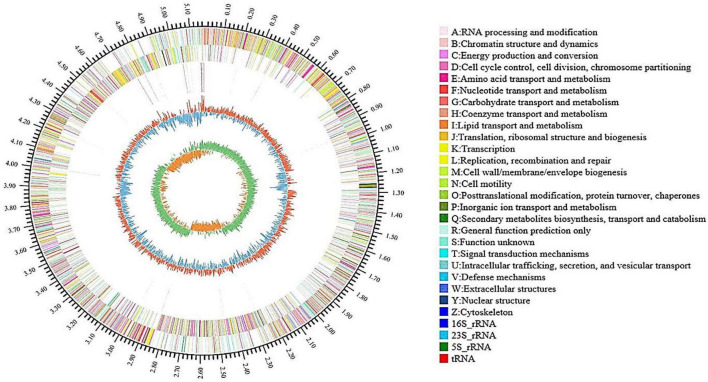
Genome map of *B. aryabhattai* TCI-16 (outside to inside): first circle shows the identification of genome information; the second and third circles are CDS distribution on positive and negative strands, and different colors indicate the different functional classification in COG categories; the fourth circle represents rRNA and tRNA; the fifth circle means the GC content, the outward part (red) indicates that the GC content of the region is higher than the average value and the inward part (blue) indicates that is lower, with higher peaks denoting a greater difference from the average GC content; the innermost circle is GC-Skew value.

As shown in Table 1, the genome of *B. aryabhattai* TCI-16 consisted of a circular chromosome, with a size of 5,167,775 bp and a GC content of 39.04%. The genome contained 5,280 protein-coding sequences with an average length of 822.90 bp, accounting for 88.37% of the entire genome. In addition, the genome included 92 rRNAs and 5 tRNAs. The complete genome of *B. aryabhattai* TCI-16 was further visualized using Circos analysis (Krzywinski et al., 2009), as shown in Figure 4.

**TABLE 1:** Statistics of gene information of *Bacillus aryabhattai* TCI-16.

Class	Number
Genome size	5,167,775bp
Total coding genes	4,344,897bp
Protein coding genes	5,280
Average length of genes	822.90bp
Gene Density	1.02 gene per kb
G + C content in Gene Region (%)	39.04
tRNA	92
rRNA	5

The predicted genes of *B. aryabhattai* TCI-16 were mapped onto the reference canonical pathways, followed by classification and functional annotation using the COG, GO, and KEGG database, respectively.

According to COG functional annotation (Supplementary Figure 1), the genes with biological functions in *B. aryabhattai* TCI-16 genome could be classified into a total of 20 types, most of which were predicted to be involved in the general function such as transcription, amino acid, carbohydrate transport and metabolism, as well as energy production and conversion. There were 276 genes involved in carbohydrate transport and metabolism, representing 6.80% of the total number of genes. The GO functional analysis of *B. aryabhattai* TCI-16 showed that a total of 2,996 genes were successfully annotated (Supplementary Figure 2), accounting for 56.74% of the total number of genes, of which 1,353, 1,464, and 2,315 genes related to biological processes, cellular components, and molecular functions, respectively.

**FIGURE 5:**
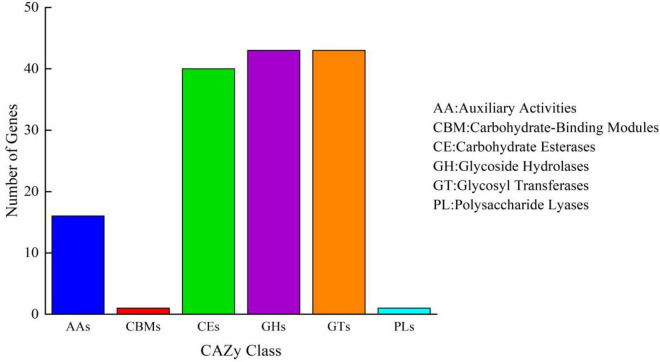
Carbohydrate-active enzyme family classification of *B. aryabhattai* TCI-16. Abscissa represents CAZy family classification and ordinate represents the number of genes.

To further identify whether *B. aryabhattai* TCI-16 had the genes responsible for encoding CDA, the predicted proteins involved in carbohydrate metabolism were annotated against the KEGG databases. A total of 2,067 functional genes were related to metabolism (Supplementary Figure 3), accounting for 72.25% of the annotated genes. Among them, there were 282 genes involved in carbohydrate metabolism, including pyruvate metabolism (ko00620), starch and sucrose metabolism (ko00500), amino sugar and nucleotide sugar metabolism (ko00520). Carbohydrate-active enzyme (CAZy) classification of *B. aryabhattai* TCI-16 was further annotated against the CAZy database, as shown in Figure 5. It was observed that a total of 17 putative genes were associated with deacetylases (Table 2), representing 11.8% of the 144 genes classified in the CAZy family. Specifically, there were 13 genes responsible for coding the CE4 (carbohydrate esterase 4) family enzymes, including one *CDA* gene coding CDA, seven *psda* genes encoding polysaccharide deacetylases, and five *pdgA* genes encoding peptidoglycan-N-acetylglucosamine deacetylases. CE4 family enzymes are metal-dependent hydrolases, which share a conserved region known as the NodB homologous domain. Still they are highly specific for their particular substrates, and exhibit diverse modes of action and mechanisms on the chitin deacetylation (Aragunde et al., 2018). CDA can specifically catalyze the N-deacetylation of chitin to generate chitosan and other physiologically active oligomers. Polysaccharide deacetylases can catalyze the hydrolysis of either the N-linked acetyl group of N-acetylglucosamine (GlcNAc) and N-acetylmuramic (MurNAc) residues or O-linked acetyl groups from O-acetylxylose residues (Andreou et al., 2018). Peptidoglycan-N-acetylglucosamine deacetylases can hydrolyze the amide linkage of the 2-N-acetyl groups of N-acetylglucosamine residues in peptidoglycan (Moynihan et al., 2014). The chitin polymer can be converted into a series of new compounds including chitosan and chitooligosaccharides *via* the depolymerization and de-N-acetylation reactions under the participation of multiple CE4 enzymes (Aragunde et al., 2018). Therefore, chitosan polysaccharides and oligosaccharides are not a single structure, which are characterized by their degree of polymerization, degree of acetylation, and pattern of acetylation. According to the whole genome annotation with regard to CAZy-family, in particular the CE4 family enzymes, it could be inferred that *B. aryabhattai* TCI-16 might be an efficient chitin-degrader owing to its abundant deacetylases.

**TABLE 2:** List of the deacetylase-related proteins identified in the *B. aryabhattai* TCI-16 genome.

	Gene name	Family	Protein length/aa	MW/kDa	pI
Chitin deacetylase	CDA	CE4	236	27.3	8.94
Polysaccharide deacetylase	psda1	CE4	326	36.9	9.30
	psda2		254	28.8	9.27
	psda3		288	33.2	8.57
	psda4		232	26.4	9.38
	psda5		294	33.0	8.67
	psda6		257	29.4	6.30
	psda7		269	31.2	9.47
Peptidoglycan-N-acetylglucosamine deacetylase	pdgA1	CE4	469	52.9	8.39
	pdgA2		206	23.8	9.18
	pdgA3		244	28.0	9.44
	pdgA4		255	29.7	8.89
	pdgA5		245	28.1	7.26
N-acetylglucosamine-6-phosphate deacetylase	nagA	CE9	394	43.0	5.44
PIG-L family deacetylase	pida	CE14	218	25.0	6.59
Bacillithiol biosynthesis deacetylase	bshB1	CE14	236	26.5	5.53
	bshB2		220	25.3	5.64

### Amino acid sequence analysis of the predicted chitin deacetylase in *Bacillus aryabhattai* TCI-16

The length of *B. aryabhattai* TCI-16 CDA (BaCDA) gene was 711 bp (the accession number deposited in GenBank: OP019603), encoding a 236-amino acid protein, and the DNA and amino acid sequences of BaCDA were shown in the Supplementary Table 1. The predicted molecular weight of BaCDA was approximately 27.3 kDa with a theoretical pI of 8.94, composed of 20 amino acids with the most abundant Leu (9.7%) and low content of Cys (0.8%). The formula of BaCDA was C_1254_H_1951_N_327_O_333_S_12_, and its total number of atoms was 3877. The extinction coefficient of BaCDA was 39545 (280 nm), and its Abs 0.1% (=1 g/L) was 1.447, assuming all pairs of Cys residues form cystines. The N-terminal of the BaCDA sequence considered was M (Met) and the estimated half-life was 30 h (mammalian reticulocytes, *in vitro*). Based on the instability index of 25.10 (less than 40) and fat index of 95.00, BaCDA was presumed to be a heat-resistant and stable protein. Moreover, the grand average of hydropathicity (GRAVY) was −0.111.

**FIGURE 6:**
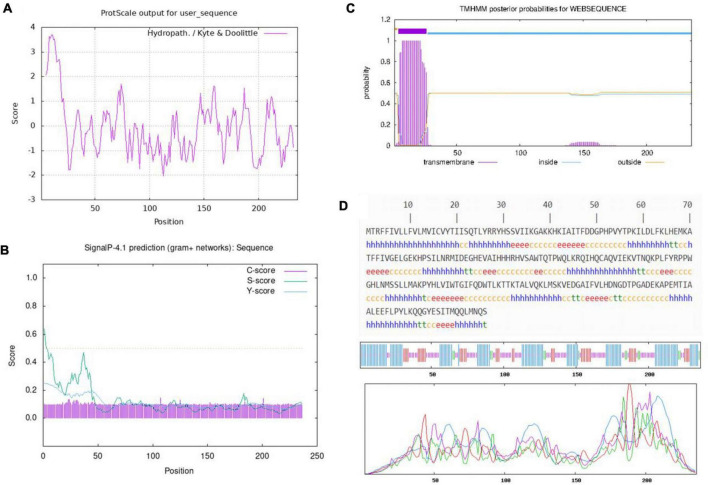
Amino acid sequence analysis of the predicted CDA. **(A)** Hydrophilicity profile of amino acid sequence of the CDA in *B. aryabhattai* TCI-16, The minimum hydrophobicity of BaCDA was −2.033, which was located at 113th amino acid (Gln); the maximum value was 3.689, which was located at 11th (Val). Overall, the protein encoded by this gene is hydrophilic. Relative weight for window edges was 100% and the weight variation model was linear. **(B)** Signal P prediction of amino acid sequence of the CDA in *B. aryabhattai* TCI-16. The prediction of the N-terminal signal peptide from the amino acid sequence shows that the protein has no signal peptide. **(C)** Transmembrane helices prediction of the CDA in *B. aryabhattai* TCI-16. **(D)** Secondary structure of the CDA in *B. aryabhattai* TCI-16. The letter h denoted the alpha-helical structure, the irregular coil structure was denoted by c, t was the beta-turn structure, and e was the extended strand.

Hydrophilicity of BaCDA was predicted utilizing the Program of ProtScale (Kyte and Doolittle, 1982). The results showed that the site of 13 (Gln) was the most hydrophilic (Score: −2.033) and 11 (Val) the most hydrophobic (Score: 3.689). Throughout the peptide chain, the hydrophilic amino acids were more evenly distributed and more numerous than the hydrophobic protein, indicating that BaCDA might be hydrophilic (Figure 6A).

SignalP 4.1 Server was applied to predict whether the first 70 amino acids of BaCDA contained signal peptide sequences, and the results showed negative (Figure 6B). The maximum C, Y, and S values were at the 23*^th^* amino acid with a score of 0.120, 11th amino acid with a score of 0.192, and 1*^th^* amino acid with a score of 0.535, respectively. The average S (0.336) and D (0.248) of the possible signal peptides was less than the threshold (0.5), indicating that the BaCDA protein had no shear site and was not a secretory protein.

The prediction results using TMHMM Server v.2.0 (Figure 6C) showed that, BaCDA had one transmembrane helix, a transmembrane amino acid number of 22.15, and a probability of 0.5 that the N-terminal was in the cytoplasm of the cell, indicating that the BaCDA protein might have a transmembrane structure domain and belong to transmembrane protein.

The secondary structure of BaCDA protein was predicted using SOPMA software, and the results (Figure 6D) showed that the protein consisted of 104 amino acids in the α-helical structure (h), 79 amino acids in the irregularly coiled structure (c), 14 amino acids in the β-turned structure (t), and 39 amino acids in the extended chain(e), which accounted for 44.07, 33.47, 5.93, and 16.53% of the secondary structure, respectively. It could be seen that the α-helix and irregular coils were the main components of the BaCDA protein structure, with the extended chain and β-turned structure dispersed in between.

**FIGURE 7:**
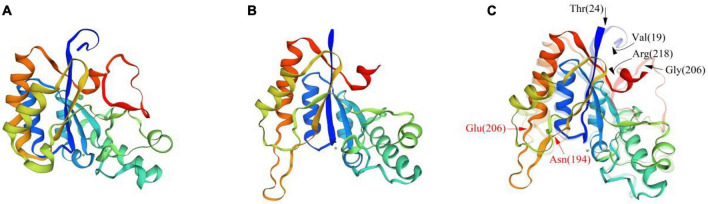
Comparison of the tertiary structures of BaCDA and ArCE4A proteins. **(A)** Tertiary structure of ArCE4A protein of *Arthrobacter* sp. (5lfz.1. A). **(B)** Tertiary structure of BaCDA protein, using the crystal structure of ArCE4A protein from *Arthrobacter* sp. (5lfz.1. A) as template sequence. **(C)** Structure comparison between BaCDA and ArCE4A. The solid part was BaCDA tertiary structure, while the translucent was ArCE4A. Arrows indicated amino acid positions with structural differences between the two.

Based on the crystal structure of ArCE4A (SMTL id 5lfz.1. A, as shown in Figure 7A), a CDA from *Arthrobacter* sp. (Tuveng et al., 2017), the homology modeling of BaCDA in *B. aryabhattai* TCI-16 was performed using SWISS-MODEL online software (Figure 7B). The result showed that BaCDA had abundant α-helices and irregular coils, and a small number of β-turns, which was consistent with the predicted secondary structure of the protein. The tertiary structures of BaCDA and ArCE4A were further superimposed and aligned, and it was found that the overall structures of the two enzymes were similar, but there were some local differences (Figure 7C). The red arrows indicated a significant difference in BaCDA structure from 194th amino acid to 206th amino acid, which contained a large number of irregularly coiled structures. The black arrows pointed to the amino acid residues in the ArCE4A tertiary structure. It could be seen that ArCE4A had long irregularly coils at the N-terminal (blue) and C-terminal (red), with the specific position from 19th amino acid to 24th amino acid and from 206th amino acid to 218th amino acid, respectively. Compared to ArCE4A, BaCDA of *B. aryabhattai* TCI-16 had shorter amino acid sequences at N-terminal and C-terminal, and this would affect the formation of multiple domains, indicating that BaCDA might possess different active sites and substrate specificity.

**FIGURE 8:**
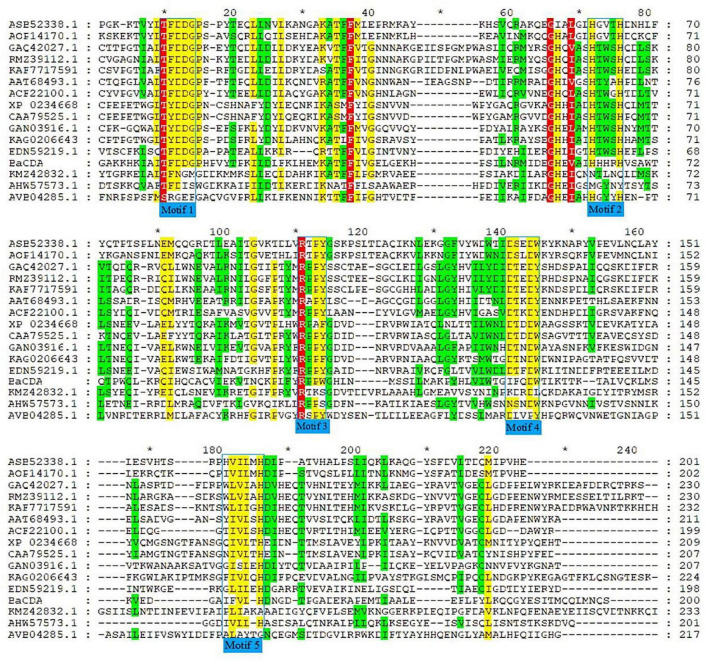
Amino alignment of CDA from several microorganisms. Amino acid sequences of CDAs from *Penicillium sp*. RFL-2021a (KAF7717591.1), *Salmonella enterica* (AVB04285.1), *Amylomyces rouxii* (CAA79525.1), *Colletotrichum lindemuthianum* (AAT68493.1), *Aspergillus niger* (GAQ42027.1), *Bacillus cereus* (AHW57573.1), *Aspergillus nidulans* (ACF22100.1), *Bacillus licheniformis* (AOP14170.1), *Mortierlla* sp. GBA30(KAG020643.1), *Aspergillus flavus* (RMZ39112.1), *Bacillus velezensis* (ASB52338.1), *Saccharomyces cerevisiae* YJM789 (EDN59219.1), *Bacillus sp.* FJAT-27238 (KMZ42832.1), *Mucor ambiguus* (GAN03916.1), and *Rhizopus microspores* ATCC52813 (XP_023466809.1) were aligned. Identical or highly conserved residues are shaded in red, while similar residues are shaded in yellow or green. The catalytic domains with five conserved motifs 1∼5 were framed by the blue box.

**FIGURE 9:**
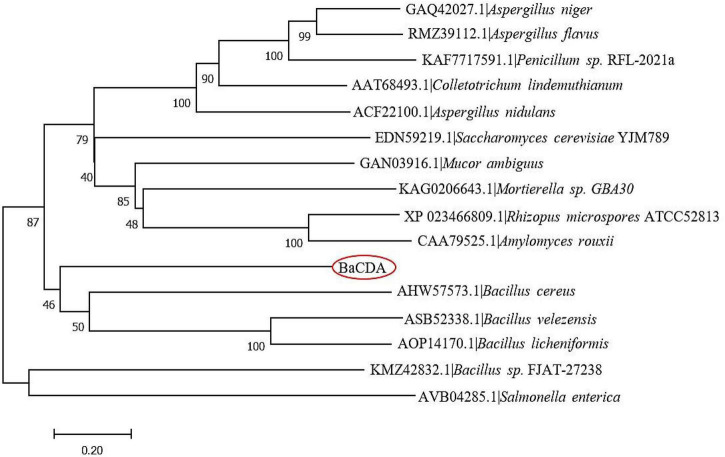
Phylogenetic tree of *B. aryabhattai* TCI-16 CDA (BaCDA) with other CDA proteins from different microorganisms based on the amino acid sequence comparisons. The evolutionary history was inferred using the Neighbor-Joining method. The optimal tree with the sum of branch length = 9.99177349 is shown.

A total of 15 CDA proteins sequences from different microbial sources were obtained on NCBI website (Wei et al., 2007; Zhu et al., 2012; Liu et al., 2015; Gong et al., 2016; Lastovetsky et al., 2016; Kuroki et al., 2017; Upadhya et al., 2018; Lenz et al., 2020), and used for multiple sequence alignment. The results showed that BaCDA shared 30.35, 31.34, and 32.56% sequence identity with the CDA of *Bacillus sp.* FJAT-27238 (KMZ42832), *Mucor ambiguus* (GAN03916.1) and *Mortierella sp.* GBA30 (KAG0206643.1), respectively. Aligned with previously defined deacetylase domains of fungal and bacterial, the catalytic domain sequences of putative BaCDA proteins were carried out using ClustalW software (Figure 8). Blair et al. (2005, 2006) described five signature motifs that made up the active sites of the deacetylase domain of CE 4 family proteins. Motifs 1–5 are represented by the sequence TFDD, H[S/T]xxH, RxP[Y/F], DxxDW, and GxxxLxH, respectively, where x is a non-specific amino acid. BaCDA shared conserved amino acid residues in motifs 1-3 with CDA from other sources. However, there were some differences in motifs 4 and 5 in the catalytic domain of BaCDA, in which motif 4 and motif 5 was replaced by IxxDW and IxxxLxH, respectively. Although the first I replaced the D in the fourth motif, the critical residue tryptophan (W), which forms one of two sides of the activity site groove, was still present. Motif 5 of the BaCDA still included a leucine (L) and a histidine (H) residue to form a hydrophobic pocket that bound the acetate methyl group (Blair et al., 2005, 2006). To investigate the evolutionary relationship among the putative BaCDA and other CDA, phylogenetic relationship analyses were carried out, and a Neighbor-Joining tree was constructed (Figure 9). It was found that BaCDA was evolutionarily close to the CDAs of the genus of *Bacillus*, but formed a separate clade.

## Discussion

There are about 10^12^–10^14^ tons of chitin generated as waste worldwide each year, and the shells of shrimp and crab in aquaculture industries are one of the primary sources of chitin (Subramanian et al., 2020). Therefore, these chitin needs to be adequately treated and degraded for efficient recovery and conversion into valuable chitosan and its derivatives. The conventional conversion process of chitin requires strong alkali and high-temperature treatment, which is a non-homogeneous reaction with random and uncontrollable deacetylation pattern, resulting in poor product stability (Lin et al., 2019). Additionally, this process is accompanied by a large amount of energy consumption, and the generated waste alkali can cause serious environmental hazards.

In contrast, enzymatic deacetylation for chitosan preparation has the advantages of cleanliness, efficiency and high specificity (Huang et al., 2022). It was reported that chitin deacetylases (CDA) could randomly bind to the long chain of chitin in multiple sites, to remove an acetyl group from the non-reducing terminal residue and then further catalyze the hydrolysis of the acetyl amine of the next long chain (Pareek et al., 2013b). Most significantly, different chitin deacetylases have different degrees of deacetylation and sites of action on chitin, and this feature is beneficial to exploit for the complex enzymatic treatment of chitin, resulting in the selective synthesis of N-acetylated oligosaccharides with high purity and unique properties (Li et al., 2007; Naqvi et al., 2016).

Mangroves, a unique type of coastal wetlands, are mainly distributed in the intertidal zone of the tropical and subtropical regions. In China, mangroves span the regions of Hainan, Guangxi, Guangdong, Fujian and Zhejiang, with a total area of 344.72 km^2^ (Zhou et al., 2020). Mangroves play essential roles in ecosystem, such as environment purifying, storing carbon and nutrients, acting as nutrient sinks for aquaculture effluents, storing pollutants, and serving as active habitats for fish, shrimp, crab and other organisms (Alongi, 2021). In addition, its particular geography, including high salinity, low oxygen, tidal gradients, high temperature, and excessive light, leads to an active microbial community. Microorganisms from mangrove soils or sediments are valuable source of metabolites with many novel chemical structures and catalytic functions. A report (Li et al., 2022) provided a comprehensive overview of 519 natural products isolated from mangrove sediments microbes and their bioactivities up to 2021, revealing that mangrove sediments microbes are an important reservoir of novel bioactive substances with considerable potential for application.

Due to mangroves being the active habitat for shrimp and crab, an oriented enrichment of CDA-producing microorganisms will probably occur in the mangroves sediments. In the present study, a strain *Bacillus aryabhattai* TCI-16 with CDA activity was successfully isolated and screened from the mangrove soil of Techeng Island in Zhanjiang, China. The fermentation characteristics of *B. aryabhattai* TCI-16 showed that the producing CDA was highly linked to cell growth, and the maximum enzyme activity could reach 120.35 ± 2.40 U/mL under shake flask culture. To gain an insight into the chitin-degrading enzyme system at genetic level, the whole genome of *B. aryabhattai* TCI-16 was sequenced and was followed by functional annotation and analysis. Function analysis of COG, GO, KEGG revealed that *B. aryabhattai* TCI-16 contained 13 putative genes responsible for coding the CE4 (carbohydrate esterase 4) family enzymes, including one gene for CDA, seven genes encoding polysaccharide deacetylases, and five genes for peptidoglycan-N-acetyl glucosamine deacetylases.

CE4 family enzymes include the *Rhizobium* NodB protein, acetylglucosamine deacetylase, chitooligosaccharides deacetylase, peptidoglycan N-acetylmuramic acid deacetylase, poly-β-1,6-N-acetylglucosamine deacetylase, and acetyl xylan esterase (Grifoll-Romero et al., 2018). These enzymes share a homologous NodB domain and contain a conserved His-His-Asp metal-binding triplet in which the metal ion adopts octahedral coordination to a conserved catalytic base (aspartic acid) and acid (histidine), which are linked to the nucleophilic reagent of three water molecules to the substrate to achieve the catalytic reaction (Blair et al., 2006; Gauthier et al., 2008; Liu et al., 2020). CDAs belong to members of CE4 family, which catalyze the N-deacetylation of chitin to form chitosan, and impart a variety of physical and chemical properties to the deacetylated chitin and participate in diverse biological functions. CDAs in plants and microorganisms are not only involved in the morphogenesis and remodeling of cell walls, but are also associated with pathogenic microbial defense escape mechanisms. CDAs of crustacean origin are able to modify the chitin structure of their cuticle and play a critical role in the molting and pupation stages of insects (Li et al., 2021).

It is well-known that bioinformatics analysis can determine the specific structural features of the target sequence and make preliminary theoretical predictions about the structure and function of the protein. The bioinformatics analyses showed that the CDA protein of *B. aryabhattai* TCI-16 was composed of 236 amino acid residues with a molecular weight of 27.3 kDa and an isoelectric point of 8.94, which was a hydrophilic, stable protein with a transmembrane structural domain. However, unlike most CDAs, the CDA in *B. aryabhattai* TCI-16 was not a secreted protein, and its secondary structure had 44.07% α-helical, 33.47% irregularly coiled, and 16.53% extended chains, respectively. Most significantly, the predicted catalytic domain of BaCDA contained five conserved motifs (Motif 1-5). Motif 1 consists of two aspartic residues, one binds metal ions to histidine residues in motif 2 to form a triplet, and the other that acts as a base to activate nucleophilic attacks on water molecules (Tuveng et al., 2017). Motifs 3 and 4 form the sides of the active groove, respectively; leucine and histidine in motif 5 form a hydrophobic substrate-binding pocket (Blair et al., 2005, 2006). The multiple sequence analysis revealed that the CDA in *B. aryabhattai* TCI-16 was different from other CDAs in amino acid sequence and size, but had highly homologous conserved catalytic domains of polysaccharide deacetylases. Moreover, phylogenetic analysis of the CDAs sequences showed that the CDA of *B. aryabhattai* TCI-16 was evolutionarily close to the CDAs of *B. cereus*, *B. licheniformis* and *B. velezensis*, but belonged to a separate clade in bacteria. In conclusion, the primary structure, spatial conformation and phylogenetic analysis suggested that the CDA of *B. aryabhattai* TCI-16 might have novel structure.

With the widespread use of chitosan in many fields, the production of chitosan by CDA enzymatic method reflects the advantages of environmental protection and controllability. In recent years, microbial derived CDAs have attracted more and more attention. He et al. (2014) reported a *B. amyloliquefaciens* strain Z7 with 18.75 U/mL of CDA activity. Ma et al. (2020) reported a CDA-producing *Rhodococcus equi* with 157.6 U/mL of CDA activity. The MeCDA from *Microbacterium esteraromaticum* MCDA02 had a molecular weight of 26 kDa, and its activity reached 137.54 U/mg (Yang et al., 2022). *Nitratireductor aquimarinus* MCDA3-3 could produce 36 kDa of NaCDA, and the enzymatic activity was 50 U/mg (Chai et al., 2020). Meanwhile, the heterologous expression of CDA has attracted a great deal of concern and interest. The CDA from *B. licheniformis* was recombinantly expressed in *E. coli* pLysS, and the crude enzyme activity reached 80–84 U/mL under LB culture conditions (Bhat et al., 2019). Similarly, Raval et al. (2017) cloned and expressed the CDA of *B. licheniformis* in *E. coli* Rosetta cells. However, some common problems existing in microbial CDAs, such as lack of high-yield strain, low catalytic efficiency, unstable enzyme production, and difficulty in purification, which limit their commercialization and industrial application.

In this study, a marine CDA-producing *B. aryabhattai* TCI-16 was isolated and screened out, and an insight into the chitin-degrading enzyme system, in particular the gene coding CDA, was demonstrated using the sequencing and analysis of complete genome sequence. However, the BaCDA of strain TCI-16 also has the problems of slow substrate conversion rate and low enzyme activity. Therefore, subsequent studies, such as a high-efficiency expression and fermentation, separation and purification, and enzymatic properties of the CDA in *B. aryabhattai* TCI-16, should be further carried out to illuminate its molecular structure and deacetylation mechanism.

## Data availability statement

The datasets presented in this study can be found in online repositories. The names of the repository/repositories and accession number(s) can be found below: GenBank:OP019603.

## Author contributions

Y-YL conducted all the experiments and wrote the manuscript. L-QY, G-HL, J-HF, and J-YP helped to conduct the experiments. K-TL and M-HT designed the experiments and revised the final manuscript. All authors contributed to the article and approved the submitted version.

## Footnotes

1
www.majorbio.com

2
http://web.expasy.org/protparam/

3
https://web.expasy.org/protscale/

4
http://www.cbs.dtu.dk/services/SignalP-4.1/

5
https://services.healthtech.dtu.dk/service.php?TMHMM-2.0

6
https://npsa-prabi.ibcp.fr/cgi-bin/npsa_automat.pl?page=npsa_sopma.html

7
http://swissmodel.expasy.org/

8
https://blast.ncbi.nlm.nih.gov/Blast.cgi
