# Supplementary material for: Isolation, characterization, and genome sequencing of a novel chitin deacetylase producing Bacillus aryabhattai TCI-16
Source: Front Microbiol. 2022 Sep 12;13:999639. doi: 10.3389/fmicb.2022.999639 (PMC9511218; doi:10.3389/fmicb.2022.999639)
Supplement: Supplementary file 1 [file Data_Sheet_1.docx]

**SUPPLEMENTARY MATERIAL**

Isolation, characterization, and genome sequencing of a novel chitin deacetylase producing *Bacillus* *aryabhattai* TCI-16

**Table S1** Nucleotide sequence and amino acid sequence of *Bacillus aryabhattai* TCI-16 CDA (BaCDA ) gene (the accession number deposited in GenBank: OP019603)

|  | Sequence |
| --- | --- |
| Protein | >gene1839 BaCDA Length = 236 aa  MTRFFIVLLFVLMVICVYTIISQTLYRRYHSSVIIKGAKKHKIAITFDDGPHPVYTPKILDLFKLHEMKATFFIVGELGEKHPSILNRMIDEGHEVAIHHHRHVSAWTQTPWQLKRQIHQCAQVIEKVTNQKPLFYRPPWGHLNMSSLLMAKPYHLVIWTGIFQDWTLKTTKTALVQKLMSKVEDGAIFVLHDNGDTPGADEKAPEMTIAALEEFLPYLKQQGYESITMQQLMNQS |
| Nucleotide | >gene1839 BaCDA Length = 711 bp  ATGACTCGTTTTTTTATTGTTTTACTTTTCGTTCTAATGGTTATATGTGTATATACCATTATTTCGCAAACCCTTTACCGTCGCTATCATTCATCAGTCATAATAAAAGGGGCAAAAAAGCATAAAATAGCTATAACATTTGACGATGGGCCTCATCCTGTTTATACGCCAAAAATTTTGGACTTGTTCAAACTGCATGAAATGAAAGCTACTTTTTTTATTGTGGGAGAACTAGGGGAAAAACACCCGTCTATTTTAAACAGGATGATTGATGAAGGACATGAAGTAGCTATACATCATCATCGTCACGTGAGTGCATGGACGCAAACACCTTGGCAATTAAAAAGACAAATTCATCAATGTGCACAGGTTATAGAGAAAGTAACAAATCAAAAGCCGCTTTTTTATCGTCCGCCGTGGGGACATTTAAATATGAGCAGTTTATTAATGGCCAAGCCATATCACCTCGTGATCTGGACAGGAATCTTTCAGGATTGGACACTAAAAACAACGAAAACGGCATTAGTTCAAAAACTAATGAGTAAAGTAGAAGATGGAGCCATTTTTGTTTTACACGATAATGGAGATACCCCGGGAGCAGATGAAAAAGCACCTGAAATGACGATTGCGGCGTTAGAAGAATTTTTACCTTACTTAAAACAGCAAGGGTATGAAAGTATAACCATGCAGCAATTGATGAATCAATCGTAA |

**
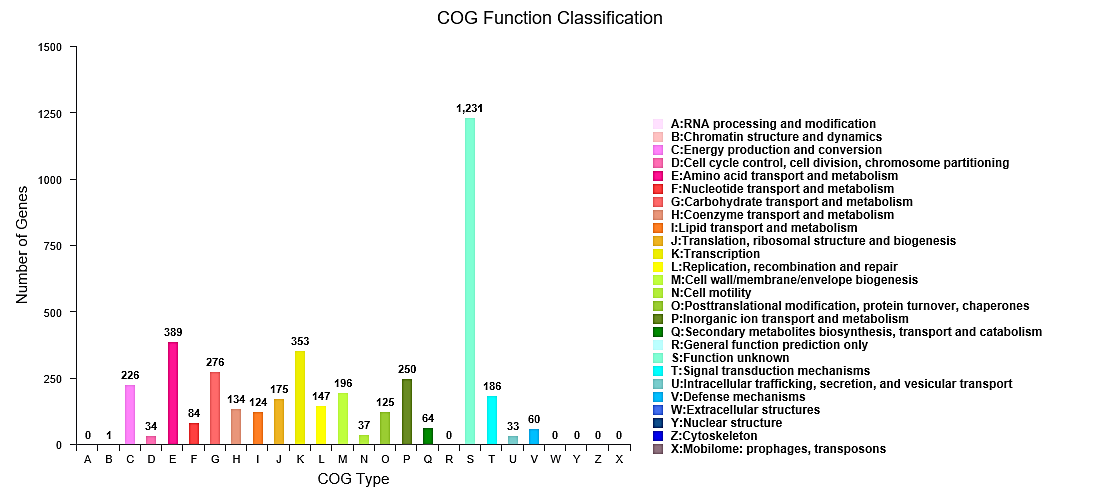
**

**Figure S1** COG functional annotations of genome of *Bacillus aryabhattai* TCI-16. Abscissa represents COG functional classification and ordinate represents the number of genes annotated within each classification.

**
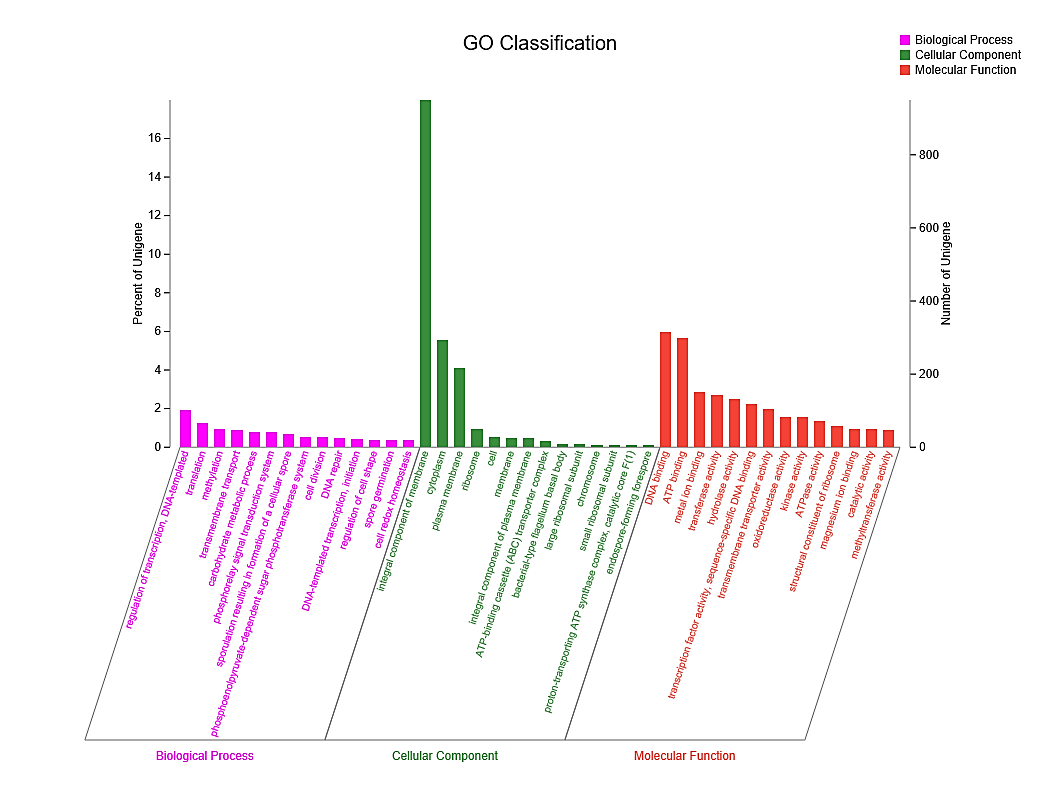
**

**Figure S2** Classification of GO functional annotations of genome of *Bacillus aryabhattai* TCI-16. A histogram showing the gene distribution of GO terms. Abscissa represents the gene number and ordinate represents GO terms. Different colours are used to distinguish biological processes, cellular components, and molecular functions.

**
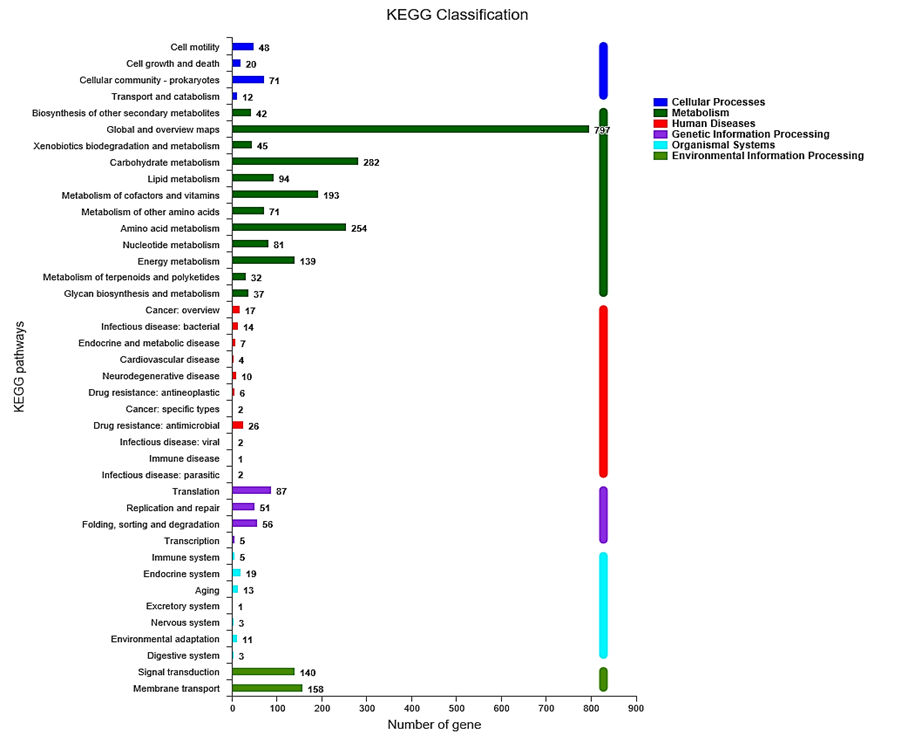
**

**Figure S3** KEGG function classification of genome of *Bacillus aryabhattai* TCI-16. KEGG function classification of genome of TCI-16. Abscissa represents the gene number and ordinate represents classification of KEGG. Different colours are used to indicate types of KEGG metabolic pathways.
